# Lower synaptic density in mood circuitry underlies depression in Parkinson’s disease

**DOI:** 10.1093/braincomms/fcag136

**Published:** 2026-04-22

**Authors:** Salih Cayir, Mika Naganawa, Tommaso Volpi, Faranak Ebrahimian Sadabad, Mark Dias, Yanghong Yang, Sophie Elliott, Mina Ansari, Amr Elshahat, Brian Pittman, Irina Esterlis, Nabeel Nabulsi, Yiyun Huang, Gerard Sanacora, Robert Comley, Sjoerd J Finnema, Richard E Carson, Sule Tinaz, David Matuskey, Sophie E Holmes

**Affiliations:** Department of Radiology and Biomedical Imaging, Yale School of Medicine, New Haven, CT 06511, USA; Department of Radiology and Biomedical Imaging, Yale School of Medicine, New Haven, CT 06511, USA; Department of Radiology and Biomedical Imaging, Yale School of Medicine, New Haven, CT 06511, USA; Department of Radiology and Biomedical Imaging, Yale School of Medicine, New Haven, CT 06511, USA; Department of Radiology and Biomedical Imaging, Yale School of Medicine, New Haven, CT 06511, USA; Department of Neurology, SUNY Downstate Health Science University, Brooklyn, NY 11203, USA; Department of Psychiatry, Yale School of Medicine, New Haven, CT 06511, USA; Department of Psychiatry, Yale School of Medicine, New Haven, CT 06511, USA; Department of Radiology and Biomedical Imaging, Yale School of Medicine, New Haven, CT 06511, USA; Department of Psychiatry, Yale School of Medicine, New Haven, CT 06511, USA; Department of Radiology and Biomedical Imaging, Yale School of Medicine, New Haven, CT 06511, USA; Department of Psychiatry, Yale School of Medicine, New Haven, CT 06511, USA; Department of Psychology, Yale School of Medicine, New Haven, CT 06511, USA; Department of Radiology and Biomedical Imaging, Yale School of Medicine, New Haven, CT 06511, USA; Department of Radiology and Biomedical Imaging, Yale School of Medicine, New Haven, CT 06511, USA; Department of Psychiatry, Yale School of Medicine, New Haven, CT 06511, USA; Discovery Research Neuroscience, Abbvie, North Chicago, IL 60085, USA; Discovery Research Neuroscience, Abbvie, North Chicago, IL 60085, USA; Department of Radiology and Biomedical Imaging, Yale School of Medicine, New Haven, CT 06511, USA; Department of Neurology, Yale School of Medicine, New Haven, CT 06511, USA; Department of Radiology and Biomedical Imaging, Yale School of Medicine, New Haven, CT 06511, USA; Department of Psychiatry, Yale School of Medicine, New Haven, CT 06511, USA; Department of Neurology, Yale School of Medicine, New Haven, CT 06511, USA; Department of Psychiatry, Yale School of Medicine, New Haven, CT 06511, USA; Department of Neurology, Yale School of Medicine, New Haven, CT 06511, USA

**Keywords:** Parkinson’s disease, depression, synaptic density, SV2A, PET

## Abstract

Depression in Parkinson’s disease is often reported as being more debilitating than the motor symptoms and has been shown to accelerate disease progression. Identifying its underlying neurobiology is crucial in the discovery of mechanism-informed treatments. We hypothesize that lower synaptic density in mood circuitry drives symptoms of depression in Parkinson’s disease. To test this hypothesis, we used PET imaging and [^11^C]UCB-J—a radiotracer that binds to synaptic vesicle protein 2A (SV2A) to image synaptic density across patients with Parkinson’s disease and depressive symptoms (PDd; *n* = 10), Parkinson’s disease patients without depressive symptoms (PDnd; *n* = 20) and healthy controls (HCs; *n* = 18). The primary outcome was binding potential (BP_ND_) in mood circuitry. Participants with PDd exhibited significantly lower synaptic density compared to HC and PDnd in the dorsolateral prefrontal cortex (dlPFC) (−22.0%, *P* < 0.001; −19.9%, *P* = 0.002), anterior cingulate cortex (ACC) (−27.9%, *P* < 0.001; −24.0%, *P* = 0.002), amygdala (−25.1%, *P* < 0.001; −18.9%, *P* = 0.006) and hippocampus (−28.1%, *P* < 0.001; −20.3%, *P* = 0.003). Synaptic density was significantly and negatively correlated with the severity of depressive symptoms across all participants with Parkinson’s disease (*n* = 30) in the dlPFC (*r* = −0.59, *P* = 0.002), ACC (*r* = −0.68, *P* < 0.001), amygdala (*r* = −0.53, *P* = 0.004) and hippocampus (*r* = −0.56, *P* = 0.003). These findings provide the first *in vivo* evidence that lower synaptic density in mood-related brain regions may contribute to depression in Parkinson’s disease. If confirmed, they would support the evaluation of interventions that target synaptic loss/induce synaptic plasticity in individuals with Parkinson’s disease and comorbid depression.

## Graphical Abstract

**Graphical Abstract:**
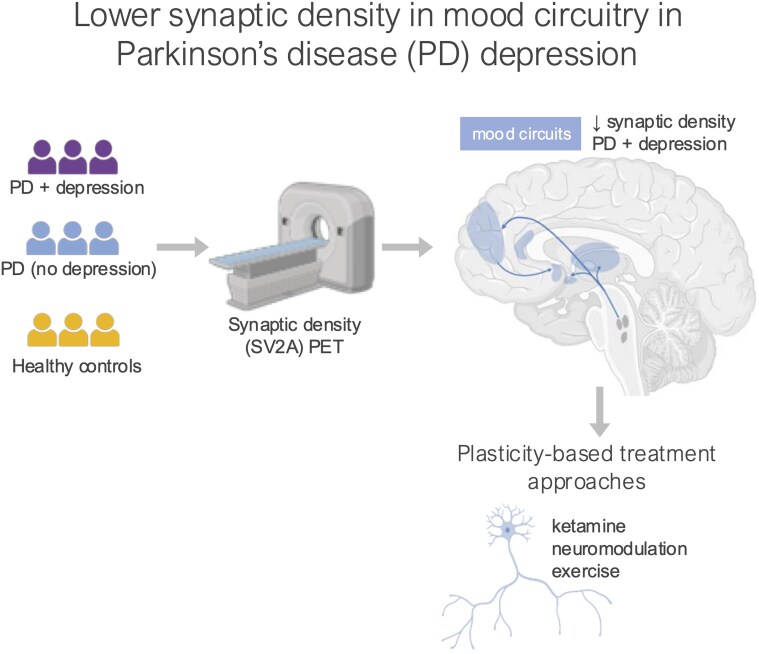

## Teaser

Cayir *et al*. report that individuals with Parkinson’s disease and depressive symptoms show lower synaptic density in mood-regulating brain circuits compared to non-depressed Parkinson’s disease patients and healthy controls. Synaptic loss in these circuits was correlated with depression severity, identifying synaptic dysfunction as a key mechanism and potential therapeutic target for depression in Parkinson’s disease.

## Introduction

Depression has a profound impact on people living with Parkinson’s disease. Affecting half of people with Parkinson’s disease, it is frequently reported as being more debilitating than the motor symptoms and, importantly, has been shown to accelerate disease progression.^1-3^ Its effective treatment is therefore critical, but serotonin-based antidepressants have limited efficacy in treating depression in Parkinson’s disease.^4,5^ This likely reflects the complex and heterogeneous pathophysiology of depression in Parkinson’s disease, involving multiple neural systems beyond serotonergic alterations. To effectively treat depression in Parkinson’s disease, it is necessary first to elucidate its underlying mechanisms.

One important clue comes from the timeline of symptom development. The characteristic motor symptoms, upon which diagnosis of Parkinson’s disease is made, are associated with a build-up of misfolded aggregates of alpha-synuclein and resultant synaptic loss in the substantia nigra (SN).^6^ However, the disease process begins decades before the onset of motor symptoms, resulting in early non-motor manifestations, including sleep disturbances, hyposmia and depression. Indeed, depression in Parkinson’s disease is thought to be driven by the presence of alpha-synuclein and resultant synaptic dysfunction beyond motor circuitry,^7^ with mounting evidence implicating disruptions within corticolimbic circuitry as a neurobiological substrate for depression in Parkinson’s disease.^8-13^

In line with this, a loss of synaptic connections and impaired synaptic function in corticolimbic circuitry—which regulates emotion processing and mood—is considered a central pathophysiological process underlying depression.^14,15^ A common pathological substrate—synaptic loss in mood circuitry—could therefore drive symptoms of depression in both major depressive disorder and Parkinson’s disease, although with distinct upstream causes. The development of the synaptic vesicle glycoprotein 2A (SV2A) radiotracer [^11^C]UCB-J by our team has introduced a non-invasive, reliable *in vivo* imaging technique for measuring synaptic density in humans via positron emission tomography (PET) imaging.^16^ We and others have leveraged this technique to show *in vivo* evidence of synaptic loss in motor circuitry in people with Parkinson’s disease.^17-19^ Indeed, we observed a direct association between synaptic density loss in the SN and the severity of motor symptoms,^20^ demonstrating that SV2A PET can effectively measure symptom-specific synaptic loss in motor circuitry in Parkinson’s disease. However, whether patients with Parkinson’s disease and depression exhibit synaptic loss within mood-related circuitry is unknown.

Here, we used SV2A PET to assess whether individuals with Parkinson’s disease and depressive symptoms show distinct lower synaptic density in corticolimbic circuitry. Uncovering this will provide much-needed new insights into the mechanisms driving depression in Parkinson’s disease and, importantly, could identify disease-specific targets to guide the development and evaluation of urgently needed, mechanism-based interventions for this prevalent, debilitating, but inadequately treated symptom of Parkinson’s disease.

## Material and methods

Thirty patients with Parkinson’s disease (diagnosed according to the Movement Disorders Society criteria^21^) and 18 healthy controls (HCs) were included in the study. The Montgomery–Åsberg Depression Rating Scale (MADRS), a 10-item clinician-rated measure of depressive symptom severity, was administered by a trained research associate under the supervision of a psychiatrist (DM). MADRS scores were used to classify Parkinson’s disease participants with (PDd, *n* = 10) and without (PDnd, *n* = 20) depressive symptoms, using the standard remission cut-off of <10.^22^ Individual MADRS scores for PDd participants are presented in Supplementary Table 1. A diagnosis of major depressive disorder was corroborated through electronic health records (EHR). Nine of the 10 PDd patients were on serotonin-based antidepressants (Supplementary Table 2). Parkinson’s disease symptoms and disease stage were assessed using the Unified Parkinson’s Disease Rating Scale (UPDRS) and Hoehn and Yahr Scale (Table 1). Participants with significant medical or neurological conditions (e.g. uncontrolled cerebrovascular disease, brain tumour, seizure disorder, dementia or any unstable medical illness that could confound interpretation of results), significant cognitive impairment [as determined by Montreal Cognitive Assessment (MoCA) score <21],^23^ a history of substance abuse, medications that affect SV2A binding (e.g. antiepileptic drugs such as levetiracetam and brivaracetam), current pregnancy or contraindications to magnetic resonance imaging (MRI) were excluded from the study. All participants were scanned and assessed in their off-medication state, after withholding dopaminergic therapy the day before per standard clinical guidelines [dopaminergic medications are reported as levodopa equivalent daily dose (LEDD); full details are provided in Supplementary Table 3]. The study was approved by the Yale University Human Investigation Committee and Yale University Radiation Safety Committee. All participants provided written informed consent.

**Table 1:** Demographic and clinical information for all participants

Variable	PDd (*n* = 10)	PDnd (*n* = 20)	HC (*n* = 18)	*P*
Age (years)^a^	63.60 ± 7.15	66.10 ± 7.95	63.04 ± 7.43	0.438
Sex (M/F)	5/5	8/12	12/6	0.220
MADRS score^a^	15.90 ± 4.53	3.70 ± 2.74	-	<0.001***
MoCA score^a^	26.10 ± 2.60	26.60 ± 2.26	-	0.534
Hoehn and Yahr Scale^b^	2.0 (0.0)	2.0 (0.0)	-	
UPDRS part III^a^	28.40 ± 11.72	28.40 ± 10.33	-	0.808
UPDRS total^a^	49.10 ± 20.68	42.75 ± 16.07	-	0.523
Parkinson’s disease duration, years^a^	5.72 ± 3.38	4.67 ± 3.12	-	0.403

^a^Data presented as mean ± SD.

^b^Data are presented as median (interquartile range). Age and sex were compared between groups using ANOVA and Fisher’s exact test, respectively. Clinical characteristics were compared between Parkinson’s disease groups using Wilcoxon rank-sum tests. Hoehn and Yahr scores were identical across participants [median (IQR) = 2.0 (0.0)]. Significance was set at *P* < 0.05, and ***indicates *P* < 0.001. Abbreviations: PDd, Parkinson’s disease with depressive symptoms; PDnd, Parkinson’s disease without depressive symptoms; HC, healthy controls; MADRS, Montgomery–Åsberg Depression Rating Scale; MoCA, Montreal Cognitive Assessment; UPDRS, Unified Parkinson’s Disease Rating Scale.

Each subject received a high-resolution three-dimensional magnetization-prepared rapid acquisition gradient echo (MPRAGE) T1-weighted scan on a 3-Tesla Siemens Prisma scanner to check for structural abnormalities and for co-registration with PET images. [^11^C]UCB-J was synthesized on-site using previously described methods.^24^ All participants underwent a single dynamic PET scan of at least 60 min using [^11^C]UCB-J on a High-Resolution Research Tomograph (Siemens), achieving a reconstructed image resolution of approximately 3 mm as detailed previously^25,26^ (Supplementary material). The primary outcome measure was non-displaceable binding potential (BP_ND_), calculated using the simplified reference tissue model 2 (SRTM2) using the centrum semiovale (CS) as the reference region^26,27^ (Supplementary Materials). Additionally, a one-tissue compartment (1TC) model (0–90 min) with a metabolite-corrected arterial plasma input function was applied to generate distribution volume (*V*_T_) for a subset of participants with available arterial input data (PDd = 6, PDnd = 13, HC = 16). The *V*_T_ of the CS was highly similar across groups (PDd, 5.25 ± 0.50 ml/cm^3^; PDnd, 5.67 ± 0.88 ml/cm^3^; HC, 5.43 ± 0.79 ml/cm^3^), with no significant group difference (*P* = 0.596). These findings support the suitability of the CS as a reference region for kinetic modelling in this cohort. Key regions within mood circuitry were chosen as primary regions of interest, including the dorsolateral prefrontal cortex (dlPFC), anterior cingulate cortex (ACC), amygdala and hippocampus. Secondary regions included motor circuitry (caudate, SN, putamen), which were assessed in exploratory analyses. All cortical and striatal regions (caudate and putamen) and the SN were grey matter segmented using SPM12 (Wellcome Trust Centre for Neuroimaging, London, UK) and corrected for partial volume effects using the Müller–Gartner (MG-PVC) algorithm.^28^ All ROIs were combined bilaterally before analyses.

## Statistical analyses

All outcomes were sufficiently normally distributed according to both Shapiro–Wilk’s tests and normality probability plots. Synaptic density was analysed using a linear mixed model with group (PDd, PDnd, HC) as a between-subjects factor and region (dlPFC, ACC, amygdala, hippocampus) as a within-subject factor. Age and sex served as covariates in all models. Duration of illness was included as an additional covariate in models excluding HC. The best-fitting variance–covariance structure was selected according to the information criteria. Residual plots confirmed model fit. Least-square (LS) means were estimated from the model, and *a priori* contrasts comparing LS means between groups within each region were performed *post hoc*. To compare continuous clinical variables (UPDRS III, UPDRS total, MADRS, MoCA and disease duration) between PDd and PDnd groups, we used Wilcoxon rank-sum tests. ANOVA and Fisher’s exact test were used to compare age and sex across all groups, respectively. The primary effect of interest was the main effect of group, which compares levels averaged over regions. False discovery rate (FDR) correction was applied to adjust for the 3 pairwise group comparisons averaged over the region and the total 12 pairwise group comparisons within each region. Potential associations between synaptic density and clinical factors among Parkinson’s disease groups were assessed using partial correlation analyses, adjusting for sex and age. Each correlation was also corrected for multiple testing using FDR for correlations between primary ROIs and MADRS. All analyses were performed using SAS (Version 9.4; SAS Institute, Cary, NC). All tests were two-sided with a significance threshold of *α* = 0.05.

## Results

### Group differences in synaptic density

**Figure 1:**
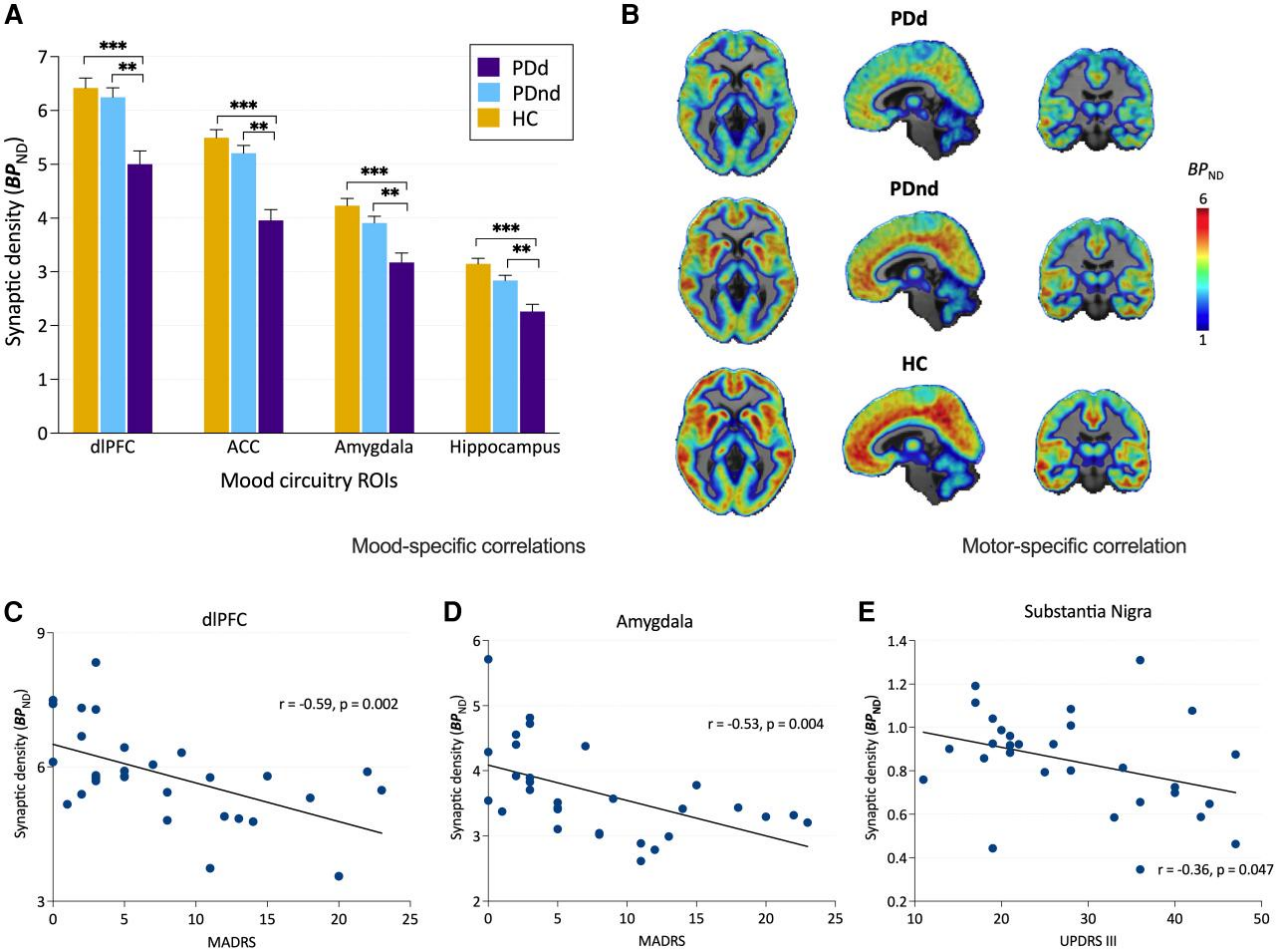
**Synaptic density differences and clinical associations in Parkinson’s disease.** (**A**) Synaptic density (SV2A BP_ND_) mean values across mood circuitry in Parkinson’s disease with depressive symptoms (PDd, *n* = 10, purple) versus without depressive symptoms (PDnd, *n* = 20, blue) and healthy controls (HC, *n* = 18, yellow). Error bars represent standard deviations. *** denotes HC > PDd at *P* < 0.001; ** denotes PDnd > PDd at *P* < 0.01. *P*-values are two-sided and FDR-adjusted (Benjamini–Hochberg) based on *post hoc* least-squares mean contrasts from a linear mixed-effects model (adjusted for age and sex) performed within each region. (**B**) Averaged parametric images of BP_ND_ values overlaid on anatomical (T1) images in MNI space. Maps show group differences in SV2A PET binding between PDd (top), PDnd (middle) and HC (bottom). The colour scale represents BP_ND_ values. (**C**, **D**) Pearson’s partial correlations (controlling for age and sex) between SV2A BP_ND_ in mood-specific regions (dlPFC and amygdala) and MADRS scores in the total Parkinson’s disease cohort (*n* = 30). Each data point corresponds to a single participant. (**E**) Pearson’s partial correlation between substantia nigra SV2A BP_ND_ and UPDRS III motor scores in the total Parkinson’s disease cohort (*n* = 30). Abbreviations: MADRS, Montgomery–Åsberg Depression Rating Scale; dlPFC, dorsolateral prefrontal cortex; ACC, anterior cingulate cortex; UPDRS III, Unified Parkinson’s Disease Rating Scale part III; ROIs, regions of interest; BP_ND_, non-displaceable binding potential.

Among primary mood regions, there was a significant main effect of group (*F*(2,43) = 18.0, *P* < 0.001) explained by lower synaptic density among PDd compared to both PDnd and HC. Participants with PDd exhibited significantly lower synaptic density compared to HC and PDnd, in the dlPFC, ACC, amygdala and hippocampus (Table 2). A significant interaction was also observed (*F*(6,135) = 3.17, *P* = 0.006), with the greatest group differences observed in the ACC (Fig. 1A, Table 2). No significant differences were observed between PDnd and HC (all *P* > 0.05). In a secondary model excluding HC, similar PDnd versus PDd effects were observed with the inclusion of disease duration as an additional covariate (data not shown). Secondary analyses were performed in motor circuitry regions for exploratory purposes (Table 2). Averaged parametric images of BP_ND_ values displaying differences between the three groups are shown in Fig. 1B.

**Table 2:** Comparison of synaptic density BP_ND_ in mood and motor circuitries between Parkinson’s disease groups and healthy controls

Region	PDd (*n* = 10)	PDnd (*n* = 20)	HC (*n* = 18)	PDd versus HC			PDd versus PDnd			PDnd versus HC		
	Mean ± SD	Mean ± SD	Mean ± SD	% diff	Cohen’s *d*	*P*	% diff	Cohen’s *d*	*P*	% diff	Cohen’s *d*	*P*
**Primary regions**												
**dlPFC**	5.00 ± 0.82	6.25 ± 0.91	6.42 ± 0.55	−22.0%	−2.15	<0.001***	−19.9%	−1.41	0.002**	−2.6%	−0.22	0.749
**ACC**	3.96 ± 0.62	5.21 ± 0.75	5.49 ± 0.51	−27.9%	−2.77	<0.001***	−24.0%	−1.76	0.002**	−5.2%	−0.44	0.377
**Amygdala**	3.17 ± 0.35	3.91 ± 0.69	4.23 ± 0.40	−25.1%	−2.79	<0.001***	−18.9%	−1.22	0.006**	−7.7%	−0.57	0.244
**Hippocampus**	2.26 ± 0.29	2.84 ± 0.51	3.15 ± 0.41	−28.1%	−2.35	<0.001***	−20.3%	−1.27	0.003**	−9.8%	−0.66	0.164
**Secondary regions**												
**Caudate**	3.68 ± 0.45	3.87 ± 0.70	4.44 ± 0.56	−17.0%	−1.44	0.025*	−4.9%	−0.30	0.382	−12.8%	−0.89	0.044*
**SN**	0.80 ± 0.28	0.87 ± 0.20	0.99 ± 0.24	−19.1%	−0.74	0.174	−7.5%	−0.29	0.382	−12.5%	−0.57	0.381
**Putamen**	4.94 ± 0.47	5.20 ± 0.80	5.45 ± 0.63	−9.3%	−0.87	0.176	−5.0%	−0.37	0.382	−4.5%	−0.34	0.382

Between-group differences were obtained by contrasting least-squares means *post hoc* within each region (PDd versus HC, PDd versus PDnd, PDnd versus HC) using a linear mixed-effects model. The model included group (PDd, PDnd, HC) and region (dlPFC, ACC, amygdala, hippocampus, caudate, substantia nigra and putamen) as factors, adjusting for age and sex. Mean and SD values represent raw, unadjusted data. Negative values indicate lower BP_ND_ in the first-listed group. *P*-values are two-sided and FDR-adjusted (Benjamini–Hochberg) within primary and secondary ROIs. Significance: *P < 0.05, P**  *<*  *0.01*, ***P < 0.001. Group sizes: PDd *n* = 10, PDnd *n* = 20, HC *n* = 18. Abbreviations: PDd, Parkinson’s disease with depressive symptoms; PDnd, Parkinson’s disease without depressive symptoms; HC, healthy controls; dlPFC, dorsolateral prefrontal cortex; ACC, anterior cingulate cortex; SN, substantia nigra; BP_ND_, binding potential.

### Synaptic density and depressive/motor symptoms

Synaptic density was negatively correlated with the severity of depressive symptoms across all patients with Parkinson’s disease (*n* = 30) in the dlPFC (*r* = −0.59, *P* = 0.002), ACC (*r* = −0.68, *P* < 0.001), amygdala (*r* = −0.53, *P* = 0.004) and hippocampus (*r* = −0.56, *P* = 0.003) (Figs. 1C and D). Further, motor severity was negatively correlated with synaptic density in the SN (*r* = −0.36, *P* = 0.047), but not in ROIs associated with mood circuitry (Fig. 1E) (Supplementary Table 4). Analyses were adjusted for sex and age.

## Discussion

Our findings provide the first direct evidence of lower synaptic density in mood circuitry in patients with Parkinson’s disease with depressive symptoms. Lower synaptic density in key regions that regulate emotions (dlPFC, ACC, amygdala and hippocampus) was associated with a greater severity of depressive symptoms across all patients with Parkinson’s disease. We also found a relationship between motor severity and synaptic density in the SN as previously reported by our group and others.^18,20^ These results reveal symptom-specific circuits, with mood and motor symptoms mapping onto distinct neural pathways.

Despite being consistently reported as one of the top factors affecting quality of life and outcomes in Parkinson’s disease, depression still lacks a clearly defined pathophysiology.^2^ Originally thought of as a reactive phenomenon and/or associated with dopamine depletion and dysfunction of the reward system, its underlying pathology was largely overlooked. However, depression is now recognized as a core manifestation of Parkinson’s disease pathology, arising from a complex interplay of alpha-synuclein accumulation, synaptic loss and neurotransmitter dysregulation.^29^ Molecular neuroimaging studies have revealed serotonergic deficits and abnormalities in dopaminergic and noradrenergic signalling within mesolimbic and corticolimbic circuits in Parkinson’s disease patients with depression.^2^ Consequently, depression in Parkinson’s disease is now understood not merely as a dysfunction of specific neurotransmitter systems or brain regions, but rather as a multidimensional disturbance affecting mood-related networks. To capture this complexity, we used SV2A PET imaging, which enables the assessment of all synapses in the brain with a single measurement. This approach accounts for the heterogeneous pathophysiology of Parkinson’s disease with depression. We observed a stepwise decline in synaptic density across groups (HC > PDnd > PDd) within mood-regulating circuitry (Fig. 1A), but not in motor regions, suggesting a coordinated relationship between lower synaptic density and depressive symptom severity in Parkinson’s disease.

Our findings have important implications for treatment and could help explain why serotonin-based antidepressants are often inadequate in Parkinson’s disease^30^—they do not directly target the putative loss of synaptic connections in mood circuitry. Rather than solely implicating serotonergic dysfunction, this likely reflects the complex pathophysiology of depression in Parkinson’s disease, which may involve disruptions in synaptic connectivity and broader mood-related neural circuits. In this context, our preliminary data implicate synapses within mood-related circuits as a biologically meaningful treatment target for novel antidepressant strategies in Parkinson’s disease. One such candidate treatment is ketamine. A substantial body of preclinical work demonstrates that ketamine exerts antidepressant-like effects through the rapid restoration of synaptic connections lost to stress and depression.^31^ In parallel, meta-analytic studies in clinical populations indicate that racemic ketamine and esketamine achieve response and remission rates in the range of approximately 60% and 30–40%, respectively, numerically comparable to first-line antidepressants, despite being tested predominantly in treatment-resistant samples.^32^ Notably, these apparent similarities in remission rates occur in the setting of markedly different patient populations and temporal response dynamics, as ketamine trials are typically conducted in treatment-resistant cohorts and are characterized by a rapid onset of antidepressant effects over hours to days, rather than the delayed, cumulative response observed with conventional monoaminergic agents. This distinction underscores ketamine’s unique mechanistic and therapeutic profile, which may be particularly relevant for mechanistically informed treatment development in Parkinson’s disease. Consistent with this framework, we previously provided initial *in vivo* human evidence that a single dose of ketamine is associated with increased synaptic density, measured using SV2A PET, within the mood circuitry of depressed patients who exhibited a synaptic deficit at baseline, suggesting that ketamine may restore lost synapses.^33^ Our findings, therefore, incentivize the evaluation of synaptogenic interventions such as ketamine in Parkinson’s disease. Further, they suggest that SV2A PET can be used to measure symptom-specific circuits, underscoring its potential as a valuable biomarker for assessing the impact of synaptogenic interventions in Parkinson’s disease. Other effective neuromodulatory antidepressant treatments, including ECT and TMS, have also been shown to induce synaptic plasticity and connectivity changes and may therefore likewise warrant mechanistically informed investigation in Parkinson’s disease.

Limitations of this pilot study include a modest sample size and participants being mildly to moderately depressed. Nevertheless, the symptom-specific correlations observed support the validity of these findings and suggest they will generalize to more severely depressed populations. A potential confound is that the majority of patients in the PDd group were taking antidepressants. However, we note that in a randomized controlled trial, the SSRI escitalopram had no significant effect on SV2A density in patients with major depressive disorder.^34^ Further, we observed no significant differences in synaptic density in patients taking antidepressants compared to the non-treatment group in our own SV2A work in major depressive disorder.^15^

This study represents a critical first step in our effort to map psychiatric symptoms to their underlying circuits and develop targeted treatments in Parkinson’s disease—an essential pursuit given the prominence of psychiatric symptoms in the world’s fastest-growing neurological disorder.^15^ Our future research will expand on this foundation by exploring the impact of synaptogenic interventions on depressive symptoms and their underlying neural circuitry in Parkinson’s disease.

## Supplementary Material

fcag136_Supplementary_Data

## Data Availability

Data are available from the corresponding author upon request. The codes used in this study are available at https://github.com/cayirsalih/Parkinsons-analysis.
