# Supplementary material for: Lower synaptic density in mood circuitry underlies depression in Parkinson’s disease
Source: Brain Commun. 2026 Apr 22;8(3):fcag136. doi: 10.1093/braincomms/fcag136 (PMC13152014; doi:10.1093/braincomms/fcag136)
Supplement: fcag136_Supplementary_Data [file fcag136_supplementary_data.docx]

**Supplementary Information**

**Title: Lower synaptic density in mood circuitry underlies depression in Parkinson’s disease**

**Short Title: Imaging Synaptic Density in PD Depression**

Salih Cayir, Mika Naganawa, Tommaso Volpi, Faranak Ebrahimian Sadabad, Mark Dias, Yanghong Yang, Sophie Elliott, Mina Ansari, Amr Elshahat, Brian Pittman, Irina Esterlis, Nabeel Nabulsi, Yiyun Huang, Gerard Sanacora, Robert Comley, Sjoerd J. Finnema, Richard E Carson, Sule Tinaz, David Matuskey, Sophie E Holmes

Sophie E Holmes and David Matuskey contributed equally to this work.

**Supplementary Material and Methods:**

**Positron Emission Tomography**

Each participant underwent a [^11^C]UCB-J PET scan, lasting up to 90 minutes, using the High-Resolution Research Tomograph (HRRT, Siemens CTI, Knoxville, TN, USA). The [^11^C]UCB-J radiotracer was synthesized on-site using established methods [1] and administered intravenously as a bolus via an automated infusion pump (Harvard PHD 22/2000, Harvard Apparatus). Prior to tracer injection, a 6-minute transmission scan was performed for attenuation correction. Head motion was continuously monitored using the Polaris Vicra optical tracking system (NDI Systems, Waterloo, Ontario, Canada). The collected data were reconstructed with corrections for attenuation, normalization, scatter, random coincidences, dead time, and motion using the MOLAR algorithm[2]. Previous studies have demonstrated that the primary outcome measure, binding potential (BP*_ND_*), can be reliably calculated without arterial blood sampling in participants with Parkinson’s disease[3] and Alzheimer’s disease[4].

**Quantitative analysis of PET data**

The primary outcome measure was BP*_ND_*, calculated using the simplified reference tissue model (SRTM2) [5]. Parametric images of BP*_ND_* were generated using the centrum semiovale as a reference region and a fixed $k_{2}^{'}$ value (clearance rate constant, *k_2_*_,_ of the reference region). A population-averaged *k_2_* of the centrum semiovale, calculated from earlier work using the one-tissue compartment model (1TCM), was used (*k_2_*=0.031±0.005 [1/min], n=17) [3, 6]. The centrum semiovale was defined using an average of individual white matter probability maps optimized to represent the core of the centrum semiovale. [6]. Additionally, we previously demonstrated that BP*_ND_*, calculated using SRTM2 with the centrum semiovale as the reference region, correlates strongly with BP*_ND_* values derived from 1TCM and arterial sampling in both PD and HC subjects[3]. Key brain regions affected in depression were chosen as the primary regions of interest (ROIs) for BP*_ND_* (Dorsolateral prefrontal cortex (dlPFC), anterior cingulate cortex (ACC), amygdala and hippocampus). As secondary analysis key motor regions affected in PD included the caudate, substantia nigra (SN), putamen. The ROIs mentioned above were defined based on the Anatomical Automatic Labeling (AAL) atlas, except for custom-drawn templates for the substantia nigra (based on previous images from a dopamine receptor tracer[7]). These ROIs were applied to the parametric images using combined transformations from the AAL template to PET space, following methods from earlier studies.

| **Supplementary Table 1. Individual Montgomery–Åsberg Depression Rating Scale (MADRS) Scores for Parkinson's Disease Patients with Depressive Symptoms.** | |
| --- | --- |
| Patient 1 | 15 |
| Patient 2 | 18 |
| Patient 3 | 12 |
| Patient 4 | 20 |
| Patient 5 | 13 |
| Patient 6 | 23 |
| Patient 7 | 11 |
| Patient 8 | 22 |
| Patient 9 | 14 |
| Patient 10 | 11 |
| Abbreviations: MADRS: Montgomery–Åsberg Depression Rating Scale; PD: Parkinson’s disease. | |

| **Supplementary Table 2**. Antidepressant Medications and Total Daily Dose for Patients with Parkinson’s Disease | | |
| --- | --- | --- |
| **Subject** | **Medication** | **mg** |
| 1 | Fluoxetine | 40 |
| 2 | Sertraline | 150 |
| 3 | Escitalopram | 20 |
| 4 | Sertraline | 200 |
| 5 | Escitalopram | 10 |
| 6 | Fluvoxamine | 50 |
| 7 | Mirtazapine | 15 |
| 8 | No Medication | N/A |
| 9 | Sertraline | 50 |
| 10 | Venlafaxine ER | 75 |
| Abbreviations: ER: extended release; mg: milligrams. | | |

| **Supplementary Table 3.** Clinical Profile of Parkinson’s Disease Medications and Levodopa Equivalent Daily Dose (LEDD) | | |
| --- | --- | --- |
| **Subject** | **PD Medication** | **LEDD,mg** |
| 1 | Amantadine; Pramipexole | 600 |
| 2 | CD/LD CR; CD/LD; Selegiline; Mirapex ER | 606 |
| 3 | Ropinirole | 80 |
| 4 | Safinamide | 100 |
| 5 | CD/LD CR; CD/LD; Ropinirole | 93 |
| 6 | CD/LD | 735 |
| 7 | CD/LD; Ropinirole | 946 |
| 8 | CD/LD; Rasagiline | 175 |
| 9 | CD/LD; Rasagiline; Amantadine | 337 |
| 10 | CD/LD; Selegiline | 300 |
| 11 | CD/LD; Amantadine; Rasagiline; Pramipexole | 563 |
| 12 | CD/LD ER; CD/LD; Amantadine; Rasagiline; | 468 |
| 13 | CD/LD; Amantadine; Rasagiline | 500 |
| 14 | CD/LD ER | 75 |
| 15 | Rasagiline | 100 |
| 16 | CD/LD ER | 75 |
| 17 | CD/LD; Ropinirole | 103 |
| 18 | No Medication | 0 |
| 19 | Amantadine; Rasagiline | 400 |
| 20 | Rasagiline | 100 |
| 21 | Rasagiline | 100 |
| 22 | Amantadine; Rasagiline | 300 |
| 23 | CD/LD; Safinamide | - |
| 24 | Rasagiline | 100 |
| 25 | CD/LD ER | 75 |
| 26 | CD/LD ER | 75 |
| 27 | CD/LD ER | 75 |
| 28 | CD/LD ER; CD/LD | 65 |
| 29 | CD/LD; Selegiline | 288 |
| 30 | CD/LD ER | 75 |
| Abbreviations: CD/LD: carbidopa/levodopa; CR: controlled release; ER: extended release; LEDD: levodopa equivalent daily dose; mg: milligrams. | | |

| **Supplementary Table 4.** Pearson’s Partial Correlation Analysis Between Clinical Measures and Synaptic Density Across Mood and Motor Circuitries. | | | | | | | | |
| --- | --- | --- | --- | --- | --- | --- | --- | --- |
|  | | **Mood Circuitry** | | | | **Motor Circuitry** | | |
| **Clinical Measures** | | **dlPFC** | **ACC** | **Amygdala** | **Hippocampus** | **Caudate** | **SN** | **Putamen** |
| **MADRS** | **r** | -0.591 | -0.684 | -0.527 | -0.561 | -0.296 | -0.097 | -0.113 |
|  | **p** | **0.002** | **<0.001** | **0.004** | **0.003** | 0.126 | 0.625 | 0.569 |
| **MoCA** | **r** | -0.076 | 0.062 | 0.086 | -0.065 | 0.053 | -0.134 | 0.112 |
|  | **p** | 0.699 | 0.752 | 0.664 | 0.743 | 0.787 | 0.495 | 0.572 |
| **UPDRS I** | **r** | -0.261 | -0.225 | -0.114 | -0.202 | -0.095 | -0.141 | 0.189 |
|  | **p** | 0.180 | 0.249 | 0.564 | 0.302 | 0.629 | 0.475 | 0.336 |
| **UPDRS II** | **r** | -0.253 | -0.185 | -0.090 | -0.079 | 0.148 | -0.474 | 0.237 |
|  | **p** | 0.193 | 0.345 | 0.649 | 0.690 | 0.452 | **0.011** | 0.225 |
| **UPDRS III** | **r** | -0.223 | -0.059 | 0.021 | 0.010 | -0.130 | -0.364 | -0.036 |
|  | **p** | 0.255 | 0.765 | 0.917 | 0.961 | 0.509 | **0.047** | 0.854 |
| **UPDRS IV** | **r** | -0.025 | -0.027 | 0.029 | -0.134 | -0.126 | 0.330 | -0.246 |
|  | **p** | 0.900 | 0.892 | 0.886 | 0.498 | 0.524 | 0.086 | 0.207 |
| **UPDRS Total** | **r** | -0.310 | -0.175 | -0.052 | -0.098 | -0.066 | -0.362 | 0.098 |
|  | **p** | 0.108 | 0.373 | 0.792 | 0.621 | 0.740 | 0.058 | 0.621 |
| **PD Disease Duration** | **r** | -0.259 | -0.120 | -0.123 | -0.100 | 0.224 | -0.405 | 0.169 |
|  | **p** | 0.183 | 0.542 | 0.532 | 0.613 | 0.252 | **0.032** | 0.391 |
| All correlations were adjusted for age and sex. Multiple comparisons across primary ROIs were corrected using the Benjamini-Hochberg False Discovery Rate (FDR). Bolded values indicate significant associations (p<0.05). Abbreviations: MADRS: Montgomery–Åsberg Depression Rating Scale; MoCA: Montreal Cognitive Assessment; UPDRS: Unified Parkinson’s Disease Rating Scale (Parts I–IV and Total score); dlPFC: dorsolateral prefrontal cortex; ACC: anterior cingulate cortex; SN: substantia nigra. | | | | | | | | |

**References:**

1. Nabulsi, N.B., J. Mercier, D. Holden, et al., *Synthesis and Preclinical Evaluation of 11C-UCB-J as a PET Tracer for Imaging the Synaptic Vesicle Glycoprotein 2A in the Brain.* J Nucl Med, 2016. **57**(5): p. 777-84.

2. Jin, X., T. Mulnix, J.D. Gallezot, and R.E. Carson, *Evaluation of motion correction methods in human brain PET imaging--a simulation study based on human motion data.* Med Phys, 2013. **40**(10): p. 102503.

3. Matuskey, D., S. Tinaz, K.C. Wilcox, et al., *Synaptic Changes in Parkinson Disease Assessed with in vivo Imaging.* Ann Neurol, 2020. **87**(3): p. 329-338.

4. Chen, M.K., A.P. Mecca, M. Naganawa, et al., *Assessing Synaptic Density in Alzheimer Disease With Synaptic Vesicle Glycoprotein 2A Positron Emission Tomographic Imaging.* JAMA Neurol, 2018. **75**(10): p. 1215-1224.

5. Wu, Y. and R.E. Carson, *Noise reduction in the simplified reference tissue model for neuroreceptor functional imaging.* J Cereb Blood Flow Metab, 2002. **22**(12): p. 1440-52.

6. Rossano, S., T. Toyonaga, S.J. Finnema, et al., *Assessment of a white matter reference region for (11)C-UCB-J PET quantification.* J Cereb Blood Flow Metab, 2020. **40**(9): p. 1890-1901.

7. Gallezot, J.D., M.Q. Zheng, K. Lim, et al., *Parametric Imaging and Test-Retest Variability of ¹¹C-(+)-PHNO Binding to D₂/D₃ Dopamine Receptors in Humans on the High-Resolution Research Tomograph PET Scanner.* J Nucl Med, 2014. **55**(6): p. 960-6.
